# Methods and Strategies for Enhancing the Performance of PQ/PMMA Photopolymers for Holographic Data Storage

**DOI:** 10.3390/polym18091053

**Published:** 2026-04-26

**Authors:** Junhui Wu, Lin Peng, Hao Wu, Ruying Xiong, Jingjun Huang, Enqiang Wu, Xiaodi Tan

**Affiliations:** 1 College of Photonic and Electronic Engineering, Fujian Normal University, Fuzhou 350117, China; 2 Information Photonics Research Center, Key Laboratory of Optoelectronic Science and for Medicine of Ministry of Education, Fujian Provincial Key Laboratory of Photonics Technology, Fujian Provincial Engineering Technology Research Center of Photoelectric Sensing Application, Fujian Normal University, Fuzhou 350117, China

**Keywords:** collinear holographic storage, PQ/PMMA, photopolymer, photosensitivity, diffraction efficiency, material modification, nanocomposite

## Abstract

With the advent of the big data era, traditional storage technologies struggle to meet the demands for long-term, secure, and cost-effective preservation of massive amounts of information. Collinear holographic storage technology has emerged as a strong contender for next-generation optical storage due to its high storage density, rapid parallel transmission, and exceptional reliability. Among various storage materials, phenanthraquinone-doped poly(methyl methacrylate) (PQ/PMMA) photopolymer has garnered significant attention for its negligible photo-induced volume shrinkage, low cost, controllable thickness, and polarization-sensitive holographic response properties. However, the material’s limited photosensitivity, low polarization response, and poor optical uniformity severely constrain its application in high-speed recording and multidimensional multiplexing holographic systems. This paper reviews the primary methods and strategies employed over the past five years to enhance the holographic performance of PQ/PMMA photopolymer materials, based on the microscopic physicochemical mechanisms underlying traditional and polarization holography, including chemical modification, nanoscale doping, mechanical control, etc. Through a systematic review of these research advances, this paper aims to provide theoretical foundations and technical references for developing high-performance PQ/PMMA photopolymer materials suitable for collinear holographic storage.

## 1. Introduction

**Figure 1:**
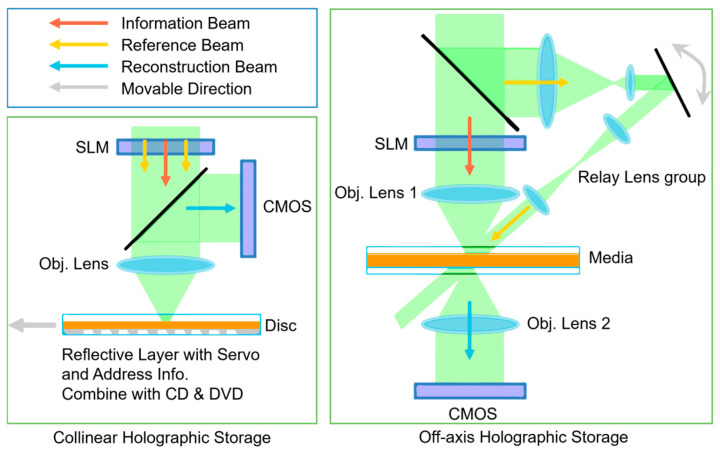
Collinear vs. off-axis holographic storage comparison.

In the current era of data explosion, the total global data volume is growing at an unprecedented rate, projected to reach 2142 ZB by 2035 [1,2,3]. Traditional magnetic storage and semiconductor storage technologies face multiple challenges regarding cost, energy consumption, and lifespan when meeting the long-term archiving needs of massive cold data [4,5,6,7,8]. Volume holographic storage, utilizing the principle of light interference, achieves a dual leap in storage density and transfer rate through its three-dimensional storage, parallel read/write capabilities, and high redundancy. It is considered a highly promising next-generation storage technology and an ideal solution for massive data archiving [9,10,11,12,13,14]. Collinear holography, compared to an off-axis holographic storage system (Figure 1), as a practical configuration, has become a crucial direction for the practical application of holographic storage due to its simple system structure and good compatibility with existing optical disc technologies [7,13,15,16].

The storage medium is the core of holographic technology, and its performance directly impacts the practical application and commercialization of holographic data storage. Holographic storage imposes stringent requirements on the Bragg condition for reading grating information, enabling the storage of multiple holograms in the same volume of material [10,17,18,19,20,21,22]. Therefore, in practical applications of volume holographic data storage, high demands are placed on the storage material: on one hand, the material needs to possess a certain thickness (typically reaching the millimeter level) to ensure a sufficiently strict Bragg selectivity, accommodating the storage of multiple holograms; on the other hand, the matrix structure of the material must be stable to avoid significant volume shrinkage during exposure, which could lead to Bragg mismatch during hologram readout and result in readout failure. Photopolymers have become a research hotspot due to their high resolution, real-time recording capability, and low cost [23,24,25,26,27,28,29,30,31]. Among these, phenanthrenequinone-doped poly(methyl methacrylate) (PQ/PMMA) [32,33,34,35,36,37,38] stands out due to its unique advantages: during the thermal polymerization stage, MMA monomers polymerize to form a solid PMMA matrix, leaving about 10% unreacted monomers; during the information recording stage, the photosensitizer PQ reacts with residual monomers or PMMA main chains under the influence of the interference light field, forming a refractive index modulated grating, thereby recording information [15,39,40,41]. Typically, the photoreaction process induces PQ to react more readily with MMA, undergoing a [4 + 2] cycloaddition reaction between C=O and C=C bonds [42,43,44,45,46,47,48,49]. The advantages of PQ/PMMA material are:

1. Minimal photopolymerization-induced volume shrinkage, ensuring accurate readout during multiplexed storage [50,51];
2. Sensitivity to light polarization, enabling multi-dimensional modulation storage [46,52,53];
3. Inexpensive raw materials and simple preparation process [54,55,56,57,58].

Leveraging the polarization sensitivity of PQ/PMMA materials enables, beyond multi-dimensional multiplexing in holographic storage, the fabrication of high-performance polarization holographic elements [59,60,61], such as polarization detectors and polarization beam splitters. These elements can simultaneously modulate the amplitude, phase, and polarization state of light—a capability that is difficult to achieve with traditional optical elements. Furthermore, compared to micro/nano optical components such as metasurfaces, they can form complex structures simply through interference exposure without the need for precision fabrication; avoid processes such as photolithography and etching, offering significant cost-reduction potential [62,63,64,65,66,67,68,69]; and can directly encode complex polarization information and spatial structures, holding promise for replacing or supplementing traditional and micro/nano polarization optical components in the future.

However, PQ/PMMA materials also possess inherent bottlenecks: the photosensitivity of traditional PQ/PMMA is low (typically below 0.3 cm/J), leading to long recording times that cannot meet the high-speed writing requirements of collinear holographic systems [54,58]; furthermore, in the field of polarization holography, the material’s polarization sensitivity needs improvement, and its photo-induced birefringence mechanism requires further elucidation [49,55,56]; moreover, manually prepared materials often exhibit poor optical uniformity across the sample, resulting in inconsistent recording performance at different locations [57,70].

**Figure 2:**
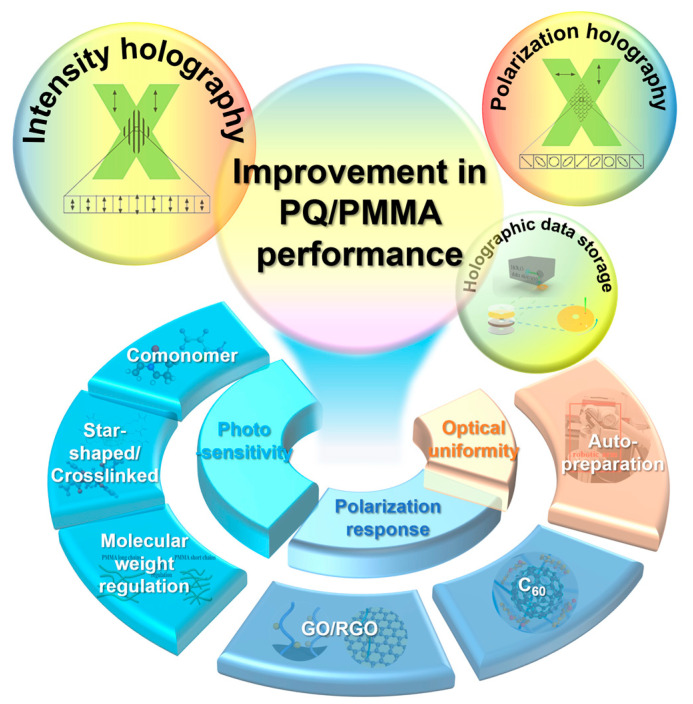
Schematic illustration of main improvement methods in PQ/PMMA.

To overcome these bottlenecks and solve the aforementioned problems, researchers worldwide, particularly the team from Fujian Normal University, have proposed various effective performance enhancement strategies based on material chemistry and physical mechanisms. A previous review by Li J. et al. detailed the development of PQ/PMMA materials before 2021, their thermal polymerization synthesis, and photopolymerization reaction mechanisms, and discussed characterization methods and strategies for improving holographic performance, such as doping with comonomers and nanoparticles [71]. In the past five years, researchers have continued in-depth research from multiple levels, including chemical synthesis [54,72,73], nanocomposite formation [49,51,56], preparation processes [70,74,75], and system adaptation [76,77,78], proposing some new insights. These include: introducing copolymerizable monomers and designing star-shaped or crosslinked network structures to regulate polymerization processes; utilizing optimized solvents to control polymer matrix molecular weight, thereby increasing residual monomers content and enhancing PQ solubility to boost material photosensitivity; incorporating nanomaterials (e.g., graphene oxide, fullerenes, reduced graphene oxide) for modification to enhance polarization properties and improve material polarization response; and developing automated fabrication platforms to enhance material optical uniformity and stability (Figure 2). Additionally, strategies for optimizing multiplexed storage performance by leveraging the material’s dark-reaction characteristics were explored. This paper aims to review and supplement these methods and strategies for enhancing the sensitivity, polarization response, and optical uniformity of PQ/PMMA photopolymers, analyze their mechanisms, further discuss the respective microscopic physicochemical mechanisms of PQ/PMMA materials in traditional holography and polarization holography, and provide a theoretical basis and technical reference for future development directions.

## 2. Recording Mechanism and Performance Evaluation Metrics of PQ/PMMA Photopolymer

### 2.1. Photoreaction and Recording Mechanism

**Figure 3:**
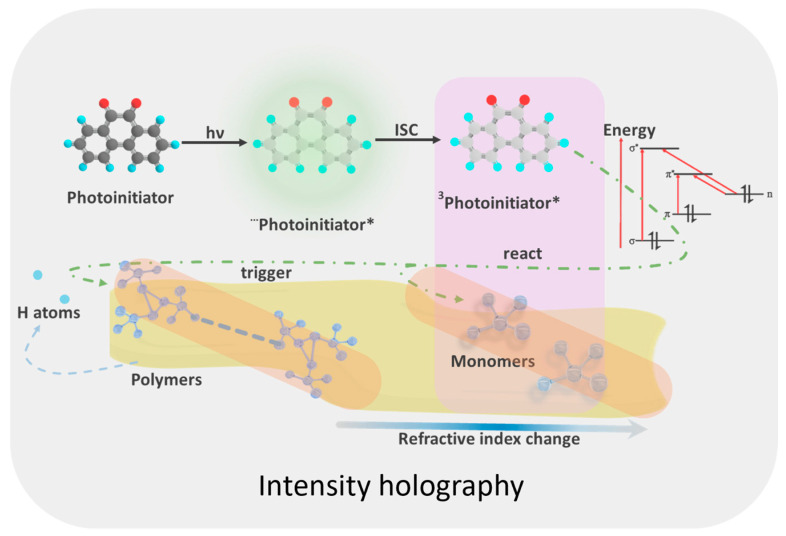
Traditional holography reaction process (“···” indicates the any singlet state of molecule, “*” indicates the excited state of molecule, and pink background frame represents the main reaction).

Holographic recording in PQ/PMMA materials is based on the synergistic effect of photopolymerization and molecular diffusion [79,80,81,82,83,84]. For traditional holography, in the bright regions of the interference pattern formed by coherent light, the photosensitizer PQ absorbs photons and transitions to an excited state, causing the electron distribution around the molecule to shift toward the terminal C=O group. At this point, it can undergo a [4 + 2] ring-opening reaction with the remaining MMA monomers to form optical products with differing refractive indices [44,47,48]. Simultaneously, it abstracts hydrogen atoms from nearby MMA monomers or PMMA chains to form radicals, which in turn trigger polymerization reactions between nearby MMA monomers and PMMA, generating high-refractive-index polymer graft products. However, since the binding energy of the reaction between PQ and MMA is lower [49], it plays a more dominant role in the photoreaction (Figure 3).

In the dark regions of the interference pattern, photoreactions scarcely occur. The resulting monomer concentration gradient between bright and dark regions drives MMA monomers to diffuse from dark regions to bright regions, while the large photoproduct molecules generated in bright regions diffuse poorly. This spatial difference in composition distribution leads to a periodic modulation of the refractive index, thus forming a stable volume holographic grating [85,86,87,88].

**Figure 4:**
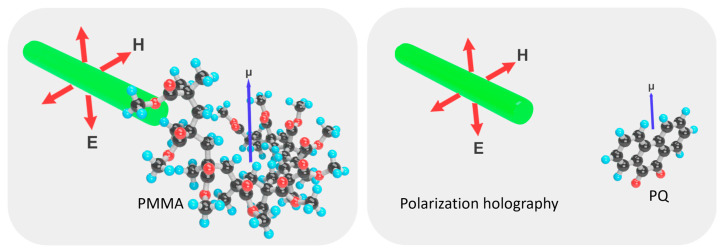
Schematic of the polarization holographic response mechanism (response of PMMA, the material with the highest polarity, and PQ, the material with the second-highest polarity, to the light field; red ball: O atom; black ball: C atom; blue ball: H atom).

For polarization holography, a clear and established reaction kinetic model is still lacking. Because the interference field in this case has no intensity variation, the change in refractive index primarily relies on the response of molecules to the electric and magnetic field components of light, followed by photo-induced aggregation to form refractive index changes [38,46,89,90,91]. This imposes certain requirements on the polarity of each component molecule. In the formed PQ/PMMA, according to Formula (1):

(1) μ=q×d

where μ is dipole moment (normal unit: Debye), a physical quantity that quantitatively describes the degree and direction of this charge separation, q is the amount of charge (difference) carried by the positive and negative charge centers of the molecule, and d is the distance between the centers of positive and negative charges [92]. The components with stronger polarity are identified as PQ and PMMA, which possess larger dipole moments (Figure 4). Concurrently, the photoreactions present in traditional intensity holography still continue to occur; PQ is excited and generates photoproducts, but the newly generated substances often have significantly reduced polarity. Therefore, performance enhancement in traditional holography is often accompanied by a further decrease in polarization response.

### 2.2. Diffraction Efficiency Evaluation Setup

**Figure 5:**
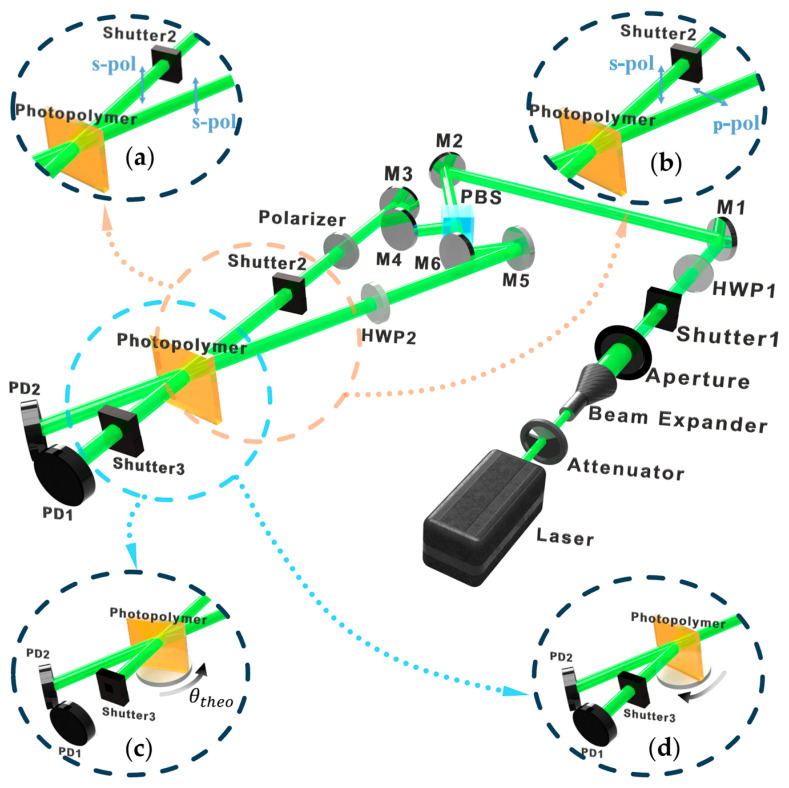
Optical response and diffraction efficiency test optical paths: (**a**) traditional holographic interference state and (**b**) orthogonal polarization interference state. Photo-induced shrinkage test: (**c**) recording process and (**d**) reading process (HWP: half-wave plate; M: reflective mirror; PBS: polarization beam splitter; PD: photo detector).

The two core metrics for evaluating the holographic performance of a material are diffraction efficiency (η) (Equation (2)) [49] and photosensitivity (S) (Equation (3)) [93]. Diffraction efficiency reflects the strength of the grating, directly impacting the signal-to-noise ratio of the readout signal; sensitivity determines the writing speed. Evaluation can be performed using the diffraction efficiency setup shown in Figure 5 [51,73]. The laser source first passes through an attenuator to control the overall light intensity, then through a beam expander, and subsequently through a half-wave plate (HWP1). When combined with a polarization beam splitter (PBS), this controls the intensity ratio of the two split beams. The polarization state of the signal beam is set to s-pol via a polarizer, while the polarization state of the reference beam is adjusted by HWP2.

(2) $$\eta =\frac{I_{1}}{I_{1}+I_{0}}$$

where $I_{1}$ and $I_{0}$ denote the Bragg-matched grating diffracted light intensity, and the light transmitted through the material can be read from the two photodetectors PD2 and PD1, respectively. This equation does not take into account factors such as the reflection of incident light at the surface of the medium, as well as the medium’s absorption and scattering of light [54,73].

(3) $$S=\frac{1}{Id}\left(\frac{\partial \sqrt{\eta }}{\partial t}\right)$$

where η is the diffraction efficiency, I is the incident light intensity, and d is the material’s thickness (normal unit: cm/J). Its physical meaning is the photosensitivity of a material per unit irradiance and per unit thickness of the medium; therefore, this equation is suitable for calculating the sensitivity of photopolymerizable materials with controllable thickness, such as PQ/PMMA [51,58]. For intensity holography testing, the reference beam is set to s-pol (Figure 5a); for polarization holography experiments, it is set to p-pol (Figure 5b). Using this optical setup, a simple fringe grating is first recorded via conventional interference inside the material to quantitatively evaluate parameters such as diffraction efficiency, photosensitivity, and transmittance (absorption) rate. Two coherent beams intersect at a symmetric angle of 24° for recording. During readout, one beam is blocked by a shutter, allowing the measurement of the diffracted light intensity from the grating at a specific moment. Accumulating data over time establishes the diffraction efficiency evolution curve during grating formation. Additionally, by changing one of the beams to a polarization state orthogonal to the other, the material’s response to polarized light can be tested. The results are also evaluated using metrics like diffraction efficiency and photosensitivity.

This same optical setup is also used to evaluate the material’s photo-induced shrinkage (volume change due to photopolymerization). This metric is crucial for holographic data storage. Significant volume shrinkage or expansion of the photopolymer material can lead to severe grating distortion, altering the Bragg condition and causing data readout errors. As shown in Figure 5c, the material is first rotated to a theoretical angle $\theta_{theo}$, and a holographic grating is recorded until the diffraction efficiency reaches its maximum. Subsequently, the material is rotated in the opposite direction while monitoring the diffracted light intensity (Figure 5d). This yields a curve of diffraction efficiency versus rotation angle. Conversion allows the determination of the central Bragg angle, denoted as $\theta_{exp}$. Finally, the shrinkage rate σ can be obtained from the calculation Equation (4) [94]:

(4) $$\sigma =1-\frac{\tan\theta_{theo}}{\tan\theta_{exp}}$$

This equation is specifically designed for cases involving asymmetric angle measurements; therefore, when testing for material shrinkage, ensuring that the material is rotated at an angle is necessary, so that a tilted grating is recorded within the material.

## 3. Constructing Efficient Initiation Systems and Reaction Networks to Improve Material Photosensitivity

Based on the mechanism of traditional holography, improving material photosensitivity fundamentally involves increasing the photoreaction rate between PQ and residual monomers. Incorporating comonomer monomers into MMA is an effective means to enhance the holographic performance of PQ/PMMA materials. This is because only a small amount of MMA monomer can react with PQ to produce photoproducts during the photoreaction. Introducing monomers with structures similar to MMA increases the number of vinyl functional groups, further enhancing the ability of monomer molecules to combine with PQ radicals to form photoproducts, thereby improving the material’s sensitivity and diffraction efficiency. Similarly, besides adding additional monomers, the content of residual monomers within the material itself can be modulated.

### 3.1. Introducing Comonomers

**Figure 6:**
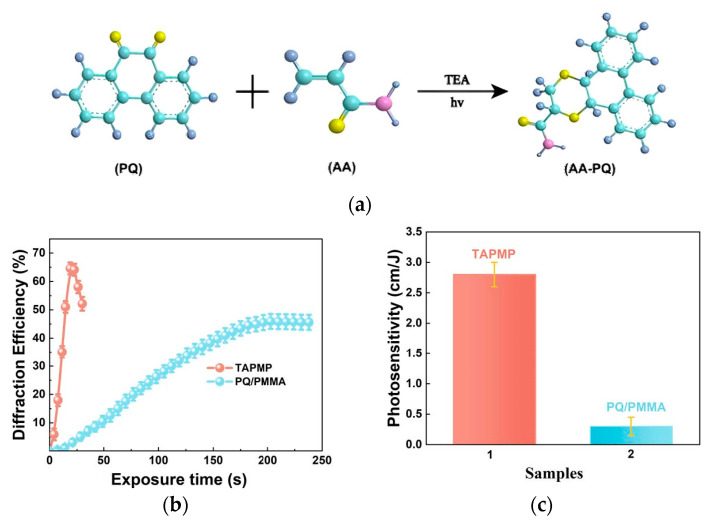
(**a**) Primary composition of TAPMP material (yellow ball: O atom; blue ball: C atom; gray ball: H atom; pink ball: N atom). (**b**) Comparison of diffraction efficiency between TAPMP material and original material (non-orthogonal interference). (**c**) Comparison of photosensitivity between TAPMP material and original material in traditional holographic recording. Reproduced with permission from ref. [72] Copyright 2022 Optica Publishing Group.

Li J. et al. prepared a novel TAPMP (TEA/AA/PQ/MBA-PMMA) material by incorporating the comonomer acrylamide (AA) [72]. Triethanolamine (TEA) was simultaneously added as an electron donor (Figure 6a), capable of undergoing electron transfer with photoexcited PQ, accelerating free radical generation. This mechanism enhanced the material’s photosensitivity by a factor of 10 (reaching 3.0 cm/J) (Figure 6c).

**Figure 7:**
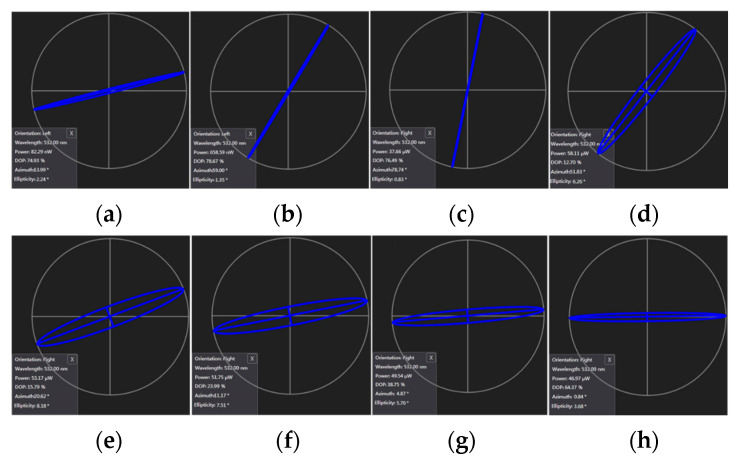
Represent the polarization state change in the diffracted light with increasing exposure time: (**a**) 20 s, (**b**) 60 s, (**c**) 100 s, (**d**) 140 s, (**e**) 180 s, (**f**) 220 s, (**g**) 260 s, (**h**) 300 s on TEA/PQ/PMMA material (The closer the blue line is to the horizontal axis, the closer the polarization state of the beam is to parallel polarization). Reproduced with permission from ref. [55] Copyright 2023 Society of Photo-Optical Instrumentation Engineers (SPIE).

Interestingly, when measuring photoinduced birefringence, they observed for the first time a “negative photoinduced birefringence” phenomenon, where the birefringence value first increased and then decreased [55]. This caused the polarization state of the diffracted light during orthogonal polarization recording to drift from the initial s-pol towards final p-pol, compromising fidelity reproduction (Figure 7). This discovery provides a new perspective for understanding the complex processes in polarization holography.

**Figure 8:**
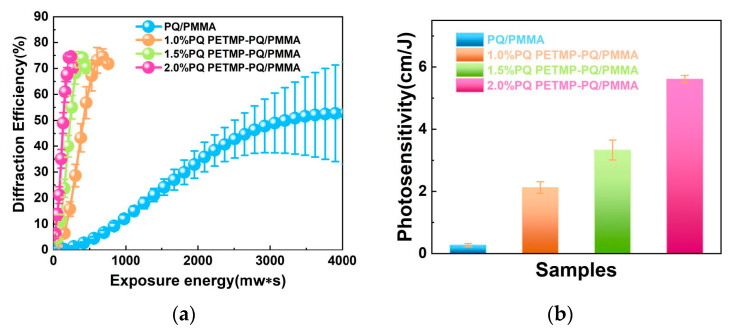
(**a**) Intensity holographic diffraction efficiency of PQ/PMMA and PETMP-PQ/PMMA with different PQ concentrations. (**b**) Photosensitivity of PQ/PMMA and PETMP-PQ/PMMA with different PQ concentrations in traditional holography. Reproduced with permission from ref. [95] Copyright 2023 Society of Photo-Optical Instrumentation Engineers (SPIE).

Zeng Z. et al., addressing the low solubility of PQ in PQ/PMMA materials, introduced the comonomer pentaerythritol tetra(3-mercaptopropionate) (PETMP), successfully increasing the PQ concentration to 2.0 wt% [95]. The photosensitivity of the new PETMP-PQ/PMMA material was improved by approximately 20 times (Figure 8b), and the diffraction efficiency increased from 50% to 75% (Figure 8a). In a collinear holographic system, the exposure time was significantly shortened, and the data bit error rate (BER) stabilized around 4%. The introduction of PETMP effectively enhanced the holographic performance of PQ/PMMA, making it suitable for high-speed holographic storage.

**Figure 9:**
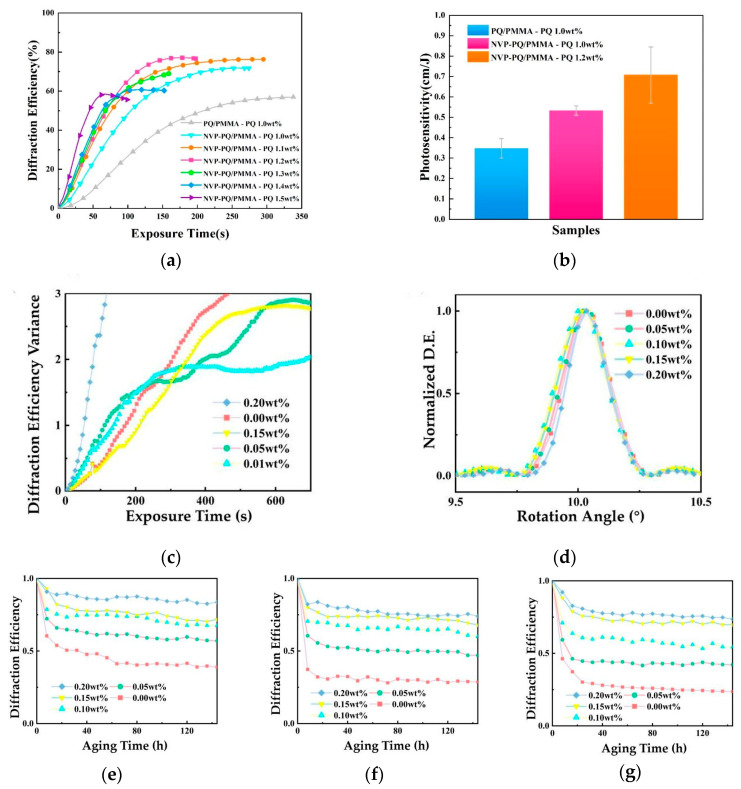
(**a**) Intensity holographic diffraction efficiency of PQ/PMMA and NVP-PQ/PMMA with different PQ concentrations. (**b**) Photosensitive histograms of PQ/PMMA and NVP-PQ/PMMA with different PQ concentrations. Reproduced with permission from ref. [96] Copyright 2023 Society of Photo-Optical Instrumentation Engineers (SPIE). (**c**) The variation in diffraction efficiency with exposure time for different NVP concentrations in traditional holography. (**d**) Rotation angle-dependent normalized diffraction efficiency of different concentrations of NVP-PQ/PMMA. Normalized diffraction efficiency of NVP-PQ/PMMA at different concentrations under aging at (**e**) 70 °C, (**f**) 75 °C, and (**g**) 78 °C. Reproduced with permission from ref. [97] Copyright 2025 Multidisciplinary Digital Publishing Institute.

Li Q. et al. introduced N-vinylpyrrolidone (NVP) into the PQ/PMMA system. Due to the better solubility of PQ in NVP, the PQ concentration was increased from 1.0 wt% to 1.2 wt% [96]. Correspondingly, the material’s photosensitivity increased from 0.35 cm/J to 0.70 cm/J (Figure 9b), and the diffraction efficiency increased from 57% to 77% (Figure 9a). Further research by Peng L. et al. showed that the introduction of NVP not only increased the concentration of C=C double bonds but also modulated the polymer molecular weight, improving material homogeneity by 38% and reducing photo-induced shrinkage by 39% (Figure 9c,d). Additionally, the introduction of NVP enhances the anti-aging performance of the holographic grating. Figure 9e,f shows the decline in diffraction efficiency at different aging temperatures; as can be seen, the grating attenuation of the modified material is significantly reduced [97].

### 3.2. Constructing Crosslinked and Star-Shaped Networks

**Figure 10:**
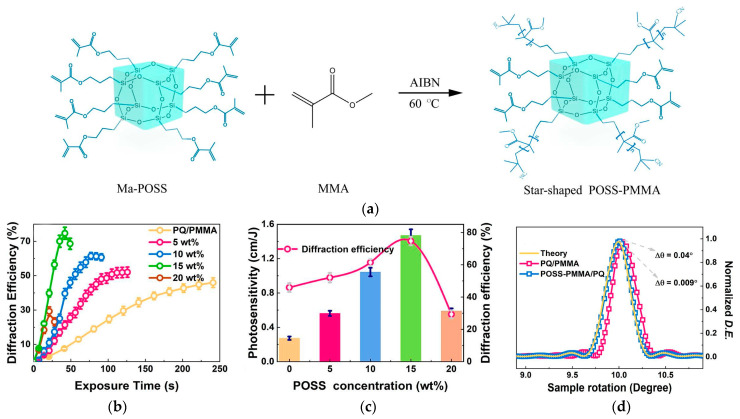
(**a**) Schematic representation of possible architecture for star-shaped POSS-PMMA macromolecules. (**b**) Time-dependent intensity holographic diffraction efficiency about PQ/PMMA and different concentrations of POSS-PMMA/PQ polymers. (**c**) POSS concentration-dependent photosensitivity and diffraction efficiency of POSS-PMMA/PQ. (**d**) Normalized diffraction efficiency of POSS-PMMA/PQ and PQ/PMMA samples that are set to be rotated for 10° from the bisector of two incidence beams as a function of the sample rotation angle. Reproduced with permission from ref. [54] Copyright 2022 American Chemical Society.

Hu P. et al. utilized eight methacryl polyhedral oligomeric silsesquioxane (Ma-POSS) with eight reactive arms to construct star-shaped POSS-PMMA macromolecules (Figure 10a) [54]. This structure, on one hand, introduced a large number of residual C=C double bonds as reactive sites, and on the other hand, acted as an internal plasticizer, loosening the polymer network and facilitating PQ diffusion. Ultimately, this achieved a 5.5-fold increase in sensitivity (1.47 cm/J) (Figure 10c) and over a 4-fold suppression of volume shrinkage (to 0.09%) (Figure 10d). This indicates that the formation of a cross-linked structure helps to further reduce the photo-induced shrinkage of the holographic polymer.

**Figure 11:**
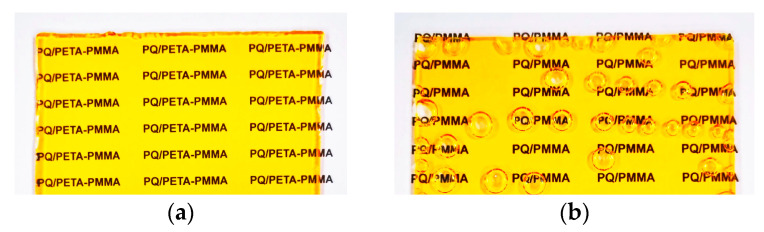

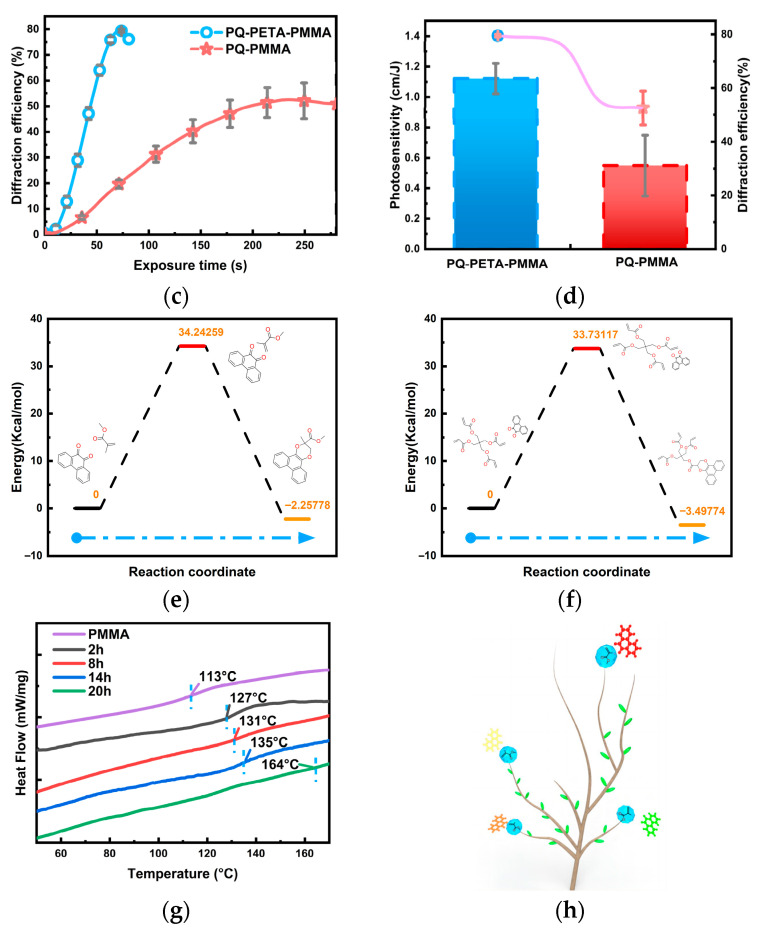
(**a**) PQ/PETA-PMMA molding case (2 h baking). (**b**) PQ/PMMA molding case (20 h baking). (**c**) Comparison between the diffraction efficiencies of PQ/PETA-PMMA and conventional PQ/PMMA. (**d**) Comparison of the optimal diffraction efficiency and photosensitivity of PQ/PETAPMMA with those of conventional PQ/PMMA. (**e**) Energy change in the PQ reaction with MMA (blue arrow indicates the direction in which the reaction occurs). (**f**) Energy change in the PQ reaction with PETA. (**g**) The glass transition temperatures of the modified materials with different curing times. (**h**) Schematic diagram illustrating the mechanism of PETA-crosslinked PQ/PMMA materials. Reproduced with permission from ref. [98] Copyright 2023 Multidisciplinary Digital Publishing Institute.

Wu J. et al. introduced the dendritic monomer pentaerythritol tetraacrylate (PETA) to construct a high-crosslinking-density network [98]. The four C=C double bonds of PETA provided abundant reactive sites, significantly shortening the material’s curing time (bubble-free samples formed in 2 h) (Figure 11a,b). The formation of the crosslinked network significantly increased the material’s glass transition temperature (T_g_) and mechanical strength (Figure 11g). Experiments combined with quantum chemical calculations indicated that the reaction energy barrier between PETA and PQ is lower than that between MMA and PQ (Figure 11e,f), leading to faster photoreaction rates. Sensitivity was doubled (1.18 cm/J) (Figure 11d), and diffraction efficiency reached 80% (Figure 11c).

**Figure 12:**
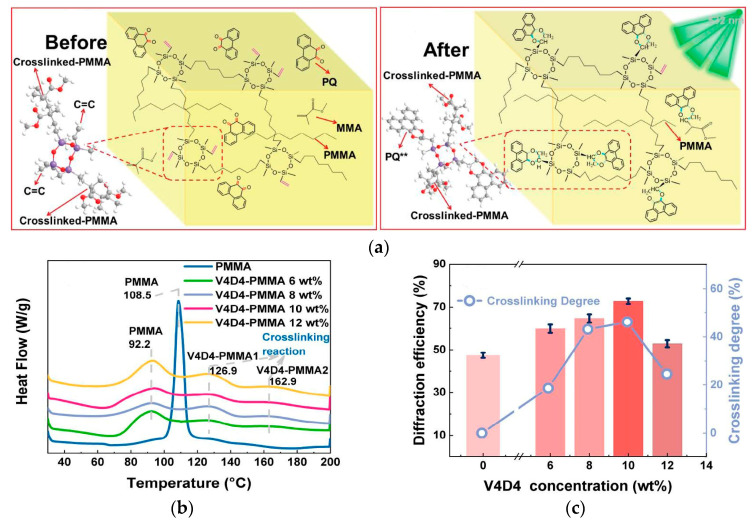
(**a**) Micromechanism diagram of photoreaction between the C=C on V4D4 and C=O on PQ photosensitizers in V4D4-PMMA/PQ matrix before and after 532 nm light exposure (“**” indicates the excited state of molecule). (**b**) Differential scanning calorimetry (DSC) heating thermograms of uncured PMMA and V4D4-PMMA materials. (**c**) Cross-linking degrees and diffraction efficiencies of PQ/PMMA and V4D4-PMMA/PQ photopolymers in traditional holography. Reproduced with permission from ref. [99] Copyright 2024 American Chemical Society.

Similarly, Hu P. et al. also introduced a 1,3,5,7-tetravinyl-1,3,5,7-tetramethylcyclotetrasi loxane (V4D4) crosslinker to construct a three-dimensional crosslinked network (Figure 12a) [99]. They revealed for the first time a strong positive correlation between the degree of crosslinking and traditional holographic performance (diffraction efficiency and sensitivity). Optimal material performance was achieved at a crosslinking degree of 46% (Figure 12c).

### 3.3. Solvent Effects and Molecular Weight Regulation

**Figure 13:**
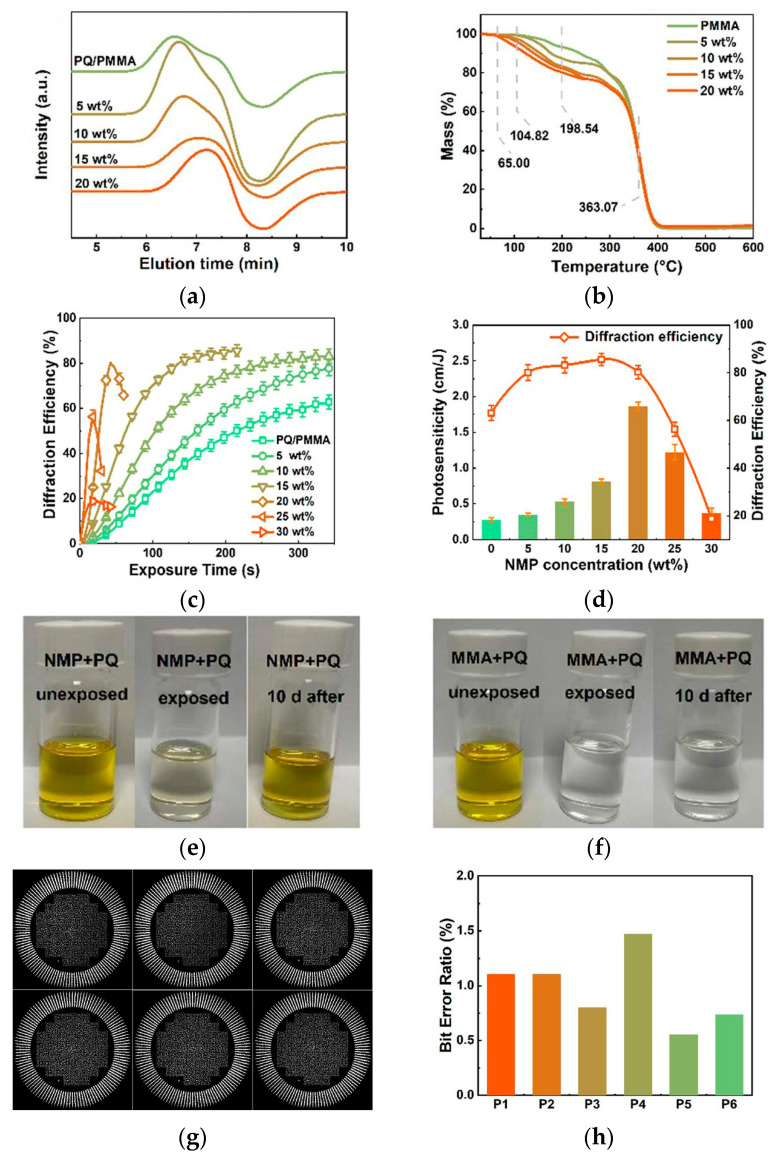
(**a**) Gel permeation chromatography (GPC) traces of NMP-PQ/PMMA at different concentrations. (**b**) Thermo gravimetric analysis (TGA) results of NMP-PMMA at different concentrations. (**c**) Exposure time-dependent diffraction efficiency of NMP-PQ/PMMA at different concentrations in traditional holography. (**d**) NMP concentration-dependent photosensitivity and diffraction efficiency of NMP-PQ/PMMA. Apparent color of the solution before exposure, after exposure, and after 10 days in the dark: (**e**) MMA + PQ and (**f**) NMP + PQ (fading caused by light exposure is usually irreversible). (**g**) A collinear holographic storage system reconstructed 2-D digital page-data images. (**h**) The BER of P1–P6. Reproduced with permission from ref. [73] Copyright 2024 Royal Society of Chemistry.

Jin J. et al. took a different approach by introducing the organic solvent N-methyl-2-pyrrolidone (NMP) to regulate the molecular weight of PMMA [73]. The study found that NMP does not participate in the thermal polymerization reaction, but its solvent effect significantly reduced the molecular weight of PMMA (from ~1.09 × 10^6^ g/mol to ~5.57 × 10^5^ g/mol) (Figure 13a) and substantially increased the content of residual MMA monomer (from 6.14% to 18.48%) (Figure 13b). More residual monomers mean more reactants for PQ, thereby enhancing photosensitivity by 6.9 times (reaching 1.86 cm/J) and increasing diffraction efficiency to over 80% (Figure 13c,d). More surprisingly, due to the reversible, weak interaction between PQ and NMP (Figure 13e,f), this material achieved at least six cycles of re-recording for the first time, holding great potential in the field of holographic information security (Figure 13g,h).

**Figure 14:**
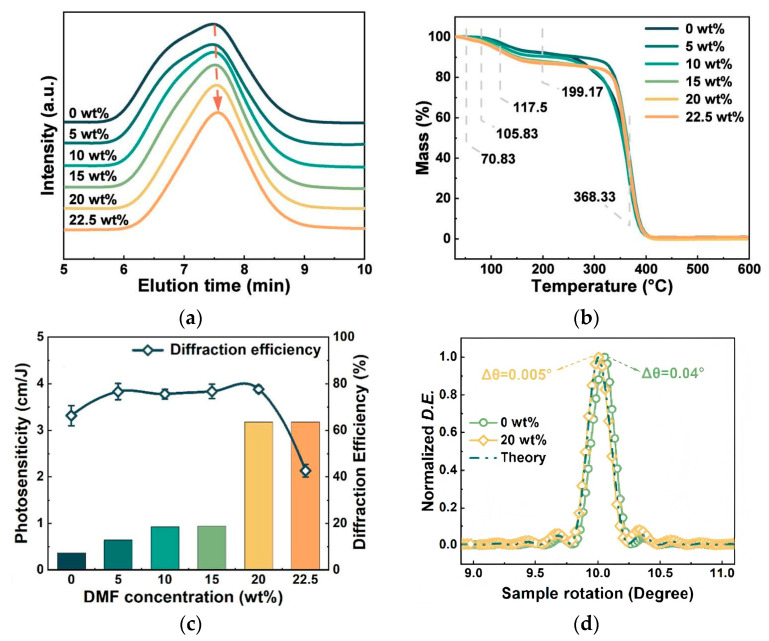
(**a**) GPC traces of different concentrations of DMG-PQ/PMMA, and (**b**) TGA results of different concentrations of DMF-PMMA. (**c**) DMF concentration-dependent photosensitivity and diffraction efficiency of DMF-PQ/PMMA. (**d**) Angular response curves for 10° holograms in 0.5 mm thick samples of PQ/PMMA and DMF-PQ/PMMA. Reproduced with permission from ref. [58] Copyright 2024 American Chemical Society.

Similarly, N,N-dimethylformamide (DMF) introduced by Jin J. et al. played an analogous role [58]. DMF acted as a plasticizer, not only modulating the molecular weight and double bond content (Figure 14a,b) but also forming hydrogen bonds with PQ molecules, further accelerating the photoreaction rate. The photosensitivity of DMF-PQ/PMMA reached a record 3.18 cm/J (a 9.1-fold increase) (Figure 14c), and the volume shrinkage rate was reduced to 0.05% (only 1/8 of the original) (Figure 14d). Although no cross-linked structure has formed here, they consider that the low shrinkage observed is due to the relatively short saturation exposure time; if the exposure time were extended to match that of conventional materials, the shrinkage might increase further.

There are also methods to improve material photosensitivity by focusing on external factors. Liu P. et al. proposed the Holographic Reciprocity Effect (HRE), describing the relationship between exposure time and the growth rate of diffraction efficiency [100]. Through nanosecond pulse and continuous-wave exposure experiments, they determined the Holographic Reciprocity Matching (HRM) range for PQ/PMMA to be 10^−6^~10^2^ s. An improved theoretical model incorporating the absorption coefficient was introduced, revealing that increasing exposure energy and modulating material thickness can avoid reciprocity failure. They suggest that HRE is a key factor affecting fast holographic storage, and controlling exposure conditions can improve reconstructed image quality.

## 4. Nanocomposite Modification to Enhance Material Polarizability

The polarization response of PQ/PMMA is directly related to the content of PQ or PMMA. To this end, nanoparticles like GO and RGO are used to reduce chain termination through physical confinement and to initiate the simultaneous growth of numerous PMMA chains via their abundant surface chemical anchor points, thereby macroscopically increasing the molecular weight of PMMA. Among these, the “grafting from” method is currently the most mainstream and efficient technique for achieving this goal [101].

### 4.1. Grafting and Sensitization with Graphene Oxide (GO)

**Figure 15:**
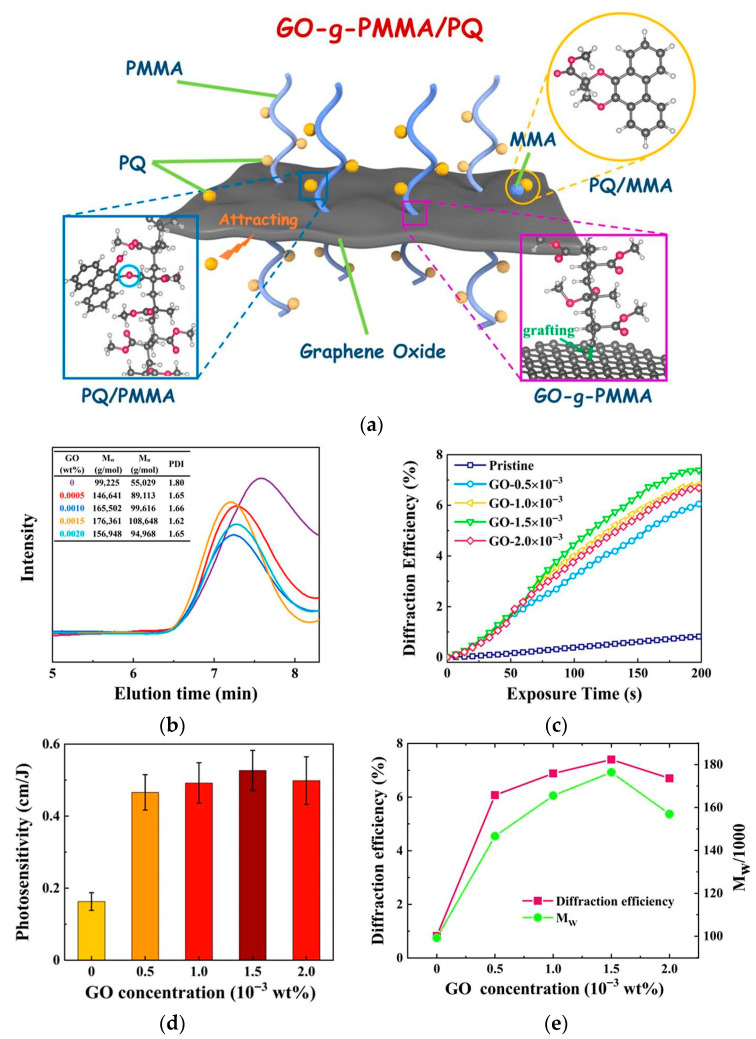
(**a**) Schematic synthesis of GO-g-PMMA/PQ. (**b**) GPC elution curves, weight-average molecular weight (M_w_), number-average molecular weight (M_n_), and polydispersity index (PDI) of GO-g-PMMA with different GO concentrations. (**c**) Exposure time-dependent orthogonal linear grating diffraction efficiency of the pristine and GO NS-doped PQ/PMMA with different concentrations. (**d**) The polarization photosensitivity of the pristine and GO NS-doped PQ/PMMA with different concentrations. (**e**) The relationship between polarization diffraction efficiency and M_w_ of GO-g-PMMA/PQ. Reproduced with permission from ref. [49] Copyright 2021 American Chemical Society.

Chen Y. et al. introduced graphene oxide (GO) into the PQ/PMMA system, pioneering the modification of photopolymers with two-dimensional materials [49]. GO can not only act as a polymerization initiator promoting MMA polymerization (Figure 15b), with its surface oxygen-containing functional groups and double bonds grafting PMMA chains to form GO-g-PMMA (Figure 15a), but also utilize its strong physical adsorption to enrich PQ molecules around the GO. This local enrichment effect, combined with the enhanced PQ solubility provided by the NMP solvent, increased the material’s orthogonal polarization diffraction efficiency by nearly 10 times (Figure 15c) and sensitivity by more than 3 times (Figure 15d). Furthermore, the relationship between molecular weight and polarization diffraction efficiency was also discovered (Figure 15e). It reveals that more PMMA will result in a higher polarization response, which is consistent with the theory.

### 4.2. Size Effect of Reduced Graphene Oxide (RGO)

**Figure 16:**
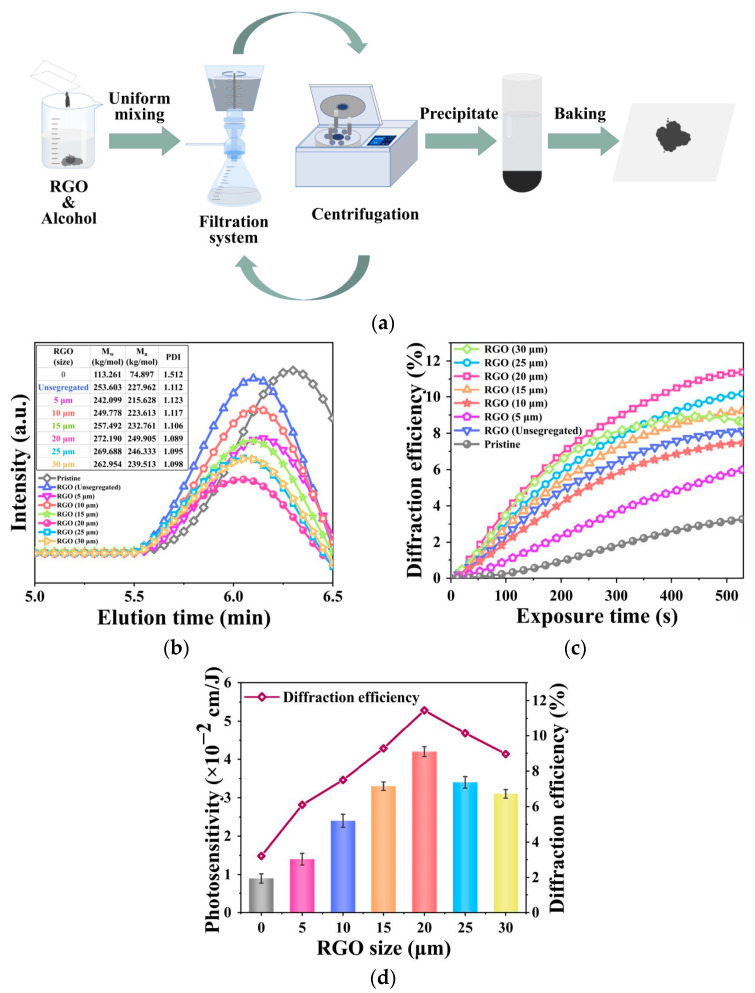
(**a**) Schematic diagram of the RGO size-grading process (arrows represent repeated manipulation). (**b**) GPC elution curves, weight average molecular weight (M_w_), number average molecular weight (M_n_), and polydispersity index (PDI) of RGO-PMMA of different sizes. (**c**) Time-dependent polarization holographic diffraction efficiencies of pristine PQ/PMMA and RGO-PMMA/PQ polymers of different sizes. (**d**) Photosensitivity and diffraction efficiency of RGO-PQ/PMMA polymers of different sizes. Reproduced with permission from ref. [56] Copyright 2023 Multidisciplinary Digital Publishing Institute.

Liu J. et al. further investigated the size effect of reduced graphene oxide (RGO) on material properties [56]. They developed a sieving technique to obtain RGO nanosheets of different sizes (5–30 μm) (Figure 16a). The study found that the size of RGO significantly influences PMMA grafting and molecular weight (Figure 16b). Sizes that were too small or too large were not conducive to performance improvement. When using 20 μm RGO, the induced RGO-PMMA exhibited the most favorable molecular weight and structure. The addition of RGO was also observed to increase the material’s molecular weight, resulting in an orthogonal polarization diffraction efficiency of 11.4% (a 3.5-fold increase) and a 4.6-fold improvement in photosensitivity (Figure 16c,d). This further confirms that the polarization response of PQ/PMMA materials most likely originates from the PMMA content.

### 4.3. Supramolecular Interactions with Fullerene (C_60_)

**Figure 17:**
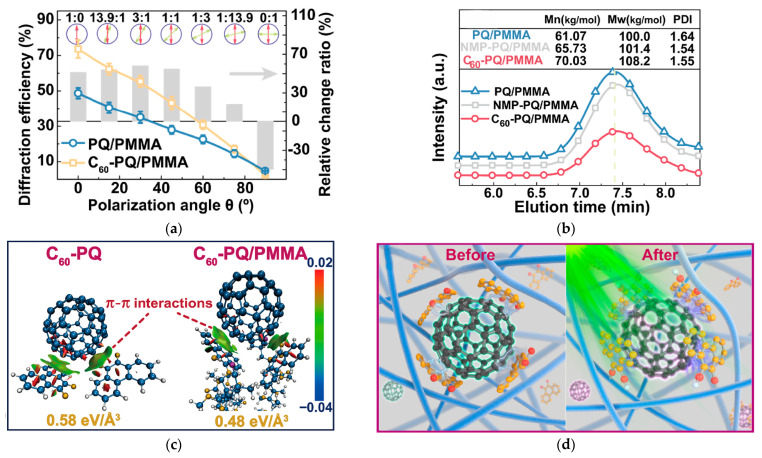
(**a**) Polarization angle-dependent saturated diffraction efficiency of PQ/PMMA and C_60_-PQ/PMMA photopolymer and corresponding relative change ratio (gray column) induced by C_60_ NPs. It also presents the changes in the polarization direction of the two beams of interference light (indicated by the red and green arrows), as well as the intensity ratio of s-pol to p-pol in the corresponding beams. (**b**) GPC evolution curve of PQ/PMMA, NMP- and C_60_-PQ/PMMA. (**c**) Schematic diagram of the weak interaction in reduced density gradient (RDG) between PQ and C_60_. These colors are specified by the color bar in the lower-left corner; the blue, red, and green colors indicate the strong attractive, strong repulsive, and van der Waals interactions, respectively. (**d**) Schematic micro-mechanism of π-π stacking interaction between C_60_ and PQ photosensitizers in PMMA matrix before and after exposure. Reproduced with permission from ref. [51] Copyright 2022 Elsevier B.V.

Hu P. et al. investigated the effect of fullerene (C_60_) on the performance of PQ/PMMA [51]. This is a typical “structure-property” relationship study. The introduction of C_60_ unexpectedly led to a “double-edged sword” effect: the diffraction efficiency for intensity holography (polarization angle is 0°) increased by over 50%, but the performance for orthogonal polarization (polarization angle is 90°) holography decreased by 49% (Figure 17a). Through spectroscopic analysis and theoretical calculations, they revealed the underlying microscopic mechanism: a strong π-π stacking interaction exists between C_60_ and PQ molecules, forming supramolecular structures with C_60_ as the core (Figure 17c,d). This structure restricts the orientation of PQ molecules during photopolymerization, thereby weakening the material’s polarization sensitivity and photoinduced anisotropy. Concurrently, C_60_ is inherently an efficient free radical scavenger, which is why it is often used as an antioxidant or radical trapper in biomedicine [102,103]. Therefore, in the free radical polymerization system of PMMA, it acts as an inhibitor, leading to no change in matrix molecular weight or even polymerization failure (Figure 17b). This finding confirms from a reverse perspective that the orientation of PQ molecules and the PMMA content are key determinants of the polarization performance of PQ/PMMA.

## 5. Optimization of Preparation Process and Polymerization Parameters to Improve Uniformity

### 5.1. Influence of Thermal Polymerization Time

**Figure 18:**
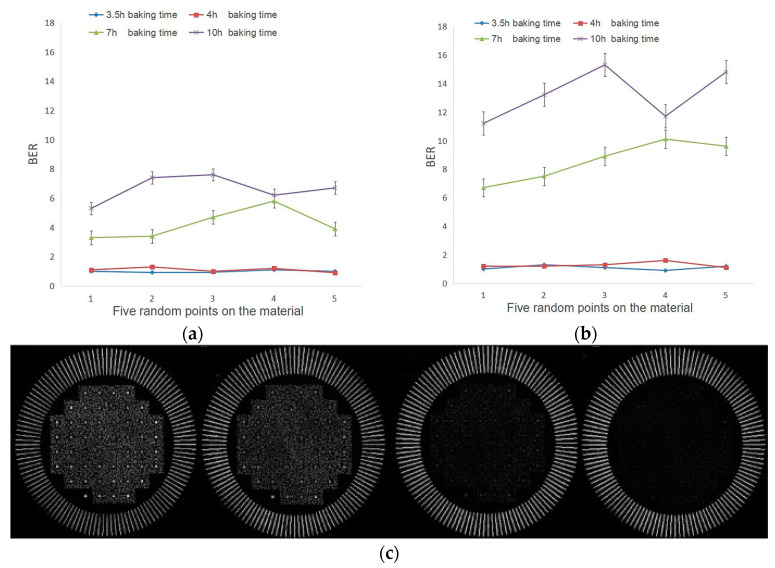

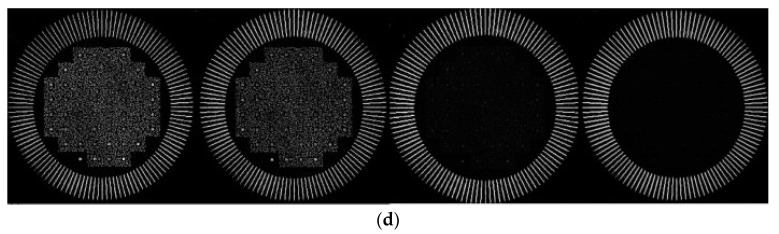
(**a**) BER of the reproduction data page at different baking times on the 1st day. (**b**) BER of the reproduction data page at different baking times on the 30th day. (**c**) Reproduction data page in the material on the 1st day at 3.5 h, 4 h, 7 h, 10 h baking time (from left to right). (**d**) Reproduction data page in the material on the 30th day at 3.5 h, 4 h, 7 h, 10 h baking time (from left to right). Reproduced with permission from ref. [74] Copyright 2022 Society of Photo-Optical Instrumentation Engineers (SPIE).

Zhang Z. et al. systematically studied the impact of thermal polymerization time on the effective usage time of the material in traditional holographic storage [74]. They found that at a polymerization temperature of 60 °C, samples cured for a shorter period (e.g., 4 h) were better able to maintain the BER at around 1% compared to those cured for a longer period (10 h) (Figure 18a,c), and their effective service life was extended by approximately 30% (Figure 18b,d). Concurrently, the diffraction efficiency was significantly improved, and the exposure time was greatly shortened. This is because, after short-term polymerization, more MMA monomers remain in the material, which can more fully react with PQ during the subsequent photopolymerization stage, forming effective refractive index modulation. Conversely, excessively long thermal polymerization times lead to the near-complete polymerization of residual monomers, not only making the material brittle but also degrading holographic performance due to the lack of reactants. This pattern is consistent with the microscopic physicochemical mechanisms of traditional holography.

### 5.2. Automated Control of the Preparation Process

**Figure 19:**
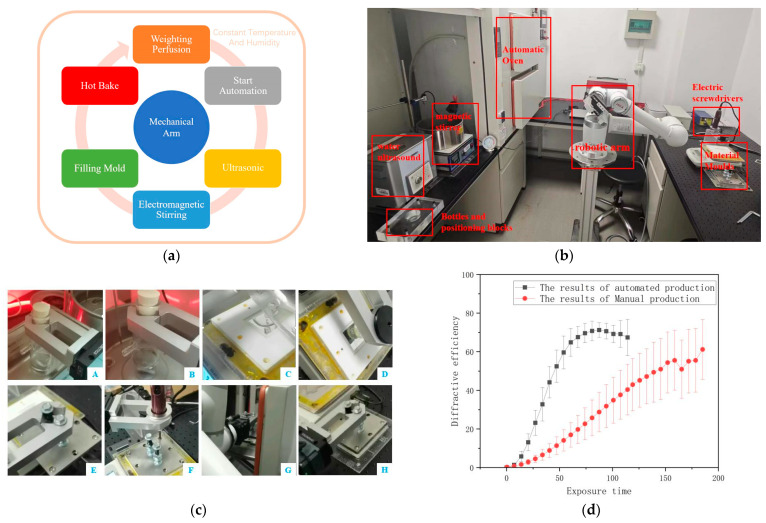
(**a**) The workflow of the automation process in an environment of constant temperature and humidity. Reproduced with permission from ref. [75] Copyright 2023 Society of Photo-Optical Instrumentation Engineers (SPIE). (**b**) Automated material room reality. Reproduced with permission from ref. [70] Copyright 2024 Society of Photo-Optical Instrumentation Engineers (SPIE). (**c**) The actual process of preparing materials through an automated system: water bath sonication (**A**), electromagnetic stirring (**B**), pour into mold (**C**), cover the glass cover (**D**), close the mold cover (**E**), screw the mold (**F**), place in automatic oven (**G**), take out the mold (**H**). (**d**) Comparison of diffraction efficiency results between manual production and automated production in traditional holography. Reproduced with permission from ref. [57] Copyright 2024 Society of Photo-Optical Instrumentation Engineers (SPIE).

Manual preparation of PQ/PMMA suffers from poor repeatability and low homogeneity. Zhang S. et al. and Yang R. et al. designed and built automated material preparation platforms (Figure 19a,b) [57,75]. Using robotic arms coupled with gravity feedback systems, they automated the entire process from ultrasonication, stirring, mold injection, to baking (Figure 19c).

**Figure 20:**
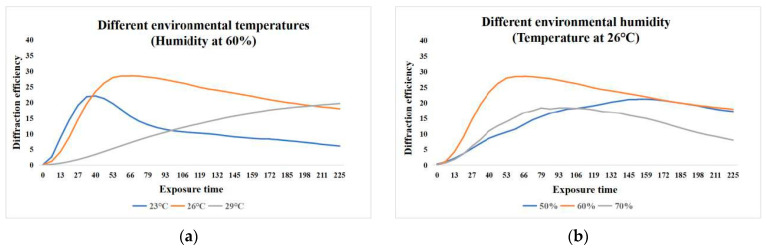
Variation in diffraction efficiency of materials with exposure time at different (**a**) temperature and (**b**) humidity environments. Reproduced with permission from ref. [70] Copyright 2024 Society of Photo-Optical Instrumentation Engineers (SPIE).

Experimental results showed that the uniformity of diffraction efficiency in automatically prepared materials was significantly superior to that in manually prepared ones. The variation in diffraction efficiency at different points was reduced from over 20% for manual preparation to within 10% (Figure 19d). This lays a solid foundation for industrial production and performance standardization of the material. Furthermore, using this platform, they investigated the influence of environmental temperature and humidity on material properties, identifying 26 °C and 60% humidity as the optimal preparation conditions (Figure 20) [70].

## 6. Exploring System-Level Adaptation and Multiplexing Strategies

### 6.1. Enhancing Shift Multiplexing Based on Dark Reactions

**Figure 21:**
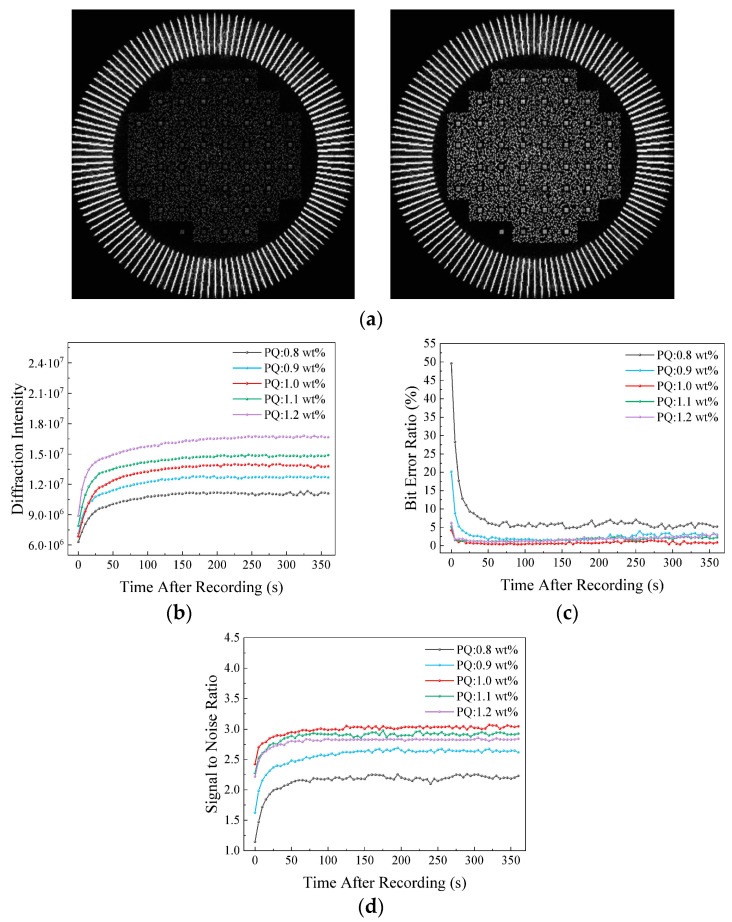
(**a**) Images without dark reaction (**left**) and 3 min after dark reaction (**right**). (**b**) Diffraction intensity, (**c**) BER, and (**d**) SNR of the reconstructed images with different PQ concentrations. Reproduced with permission from ref. [76] Copyright 2024 Society of Photo-Optical Instrumentation Engineers (SPIE).

Utilizing the principle of diffusion following traditional holographic exposure, Zhong L. et al. investigated the impact of dark reactions on reconstructed image quality at different PQ concentrations (0.8 wt%~1.2 wt%) [76]. Experiments revealed that after dark reactions at 1 wt%, diffraction intensity increased (Figure 21b), BER decreased (Figure 21c), and the signal-to-noise ratio (SNR) improved (Figure 21d). PQ at 1 wt% maintained a low BER and high signal-to-noise ratio after the dark reaction. An appropriate concentration of PQ can enhance image quality during dark reactions, with 1 wt% being the optimal concentration.

**Figure 22:**
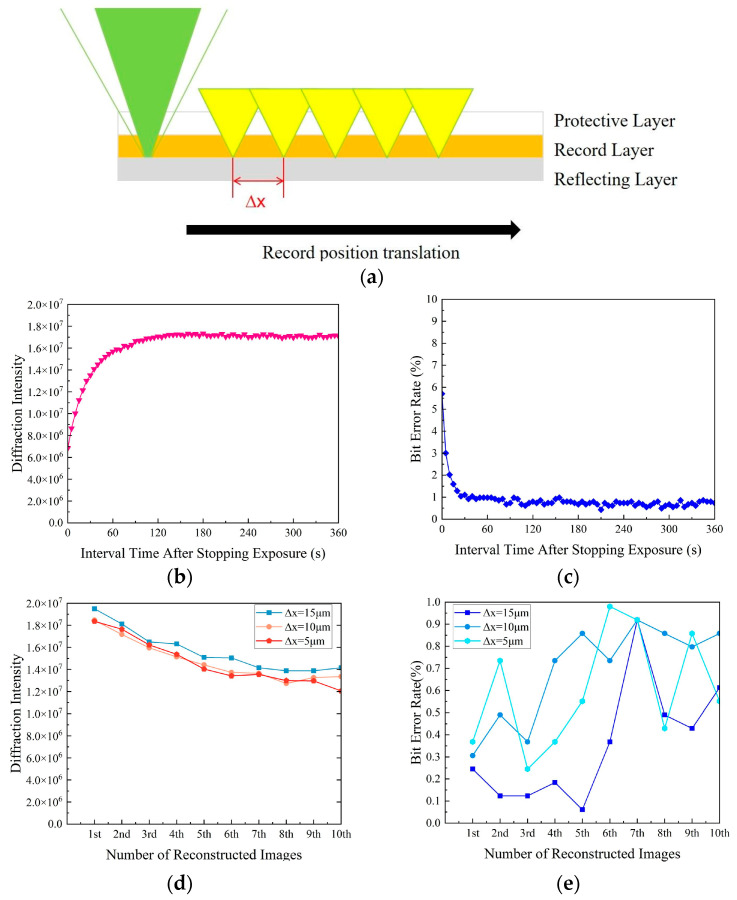
(**a**) Record point displacement on the media (green represents the recording light, and yellow indicates the multiplexing position). (**b**) Time variation curve of diffraction intensity and (**c**) bit error rate of a single hologram after stopping exposure. (**d**) Diffraction intensity and (**e**) BER of reconstructed images at three different × values after stopping exposure for 1 min dark reaction. Reproduced with permission from ref. [77] Copyright 2024 Society of Photo-Optical Instrumentation Engineers (SPIE).

Subsequently, He C. et al. cleverly utilized the “dark reaction” phenomenon of PQ/PMMA materials—where the grating further enhances in the dark after exposure ceases due to molecular diffusion [77]. In shift multiplexing experiments (Figure 22a), by allowing a 1 min rest period after exposure (dark reaction stabilization time), weak multiplexed holograms that originally exhibited high bit error rates were significantly enhanced (Figure 22b,c). At a shift step of 5 μm, the bit error rates of 10 multiplexed holograms were all reduced to below 1% (Figure 22e). This strategy, without altering the material formulation, achieves reduced multiplexing distance and improved storage quality by optimizing the system writing process.

### 6.2. Relationship Between Material Thickness and Holographic Performance

**Figure 23:**
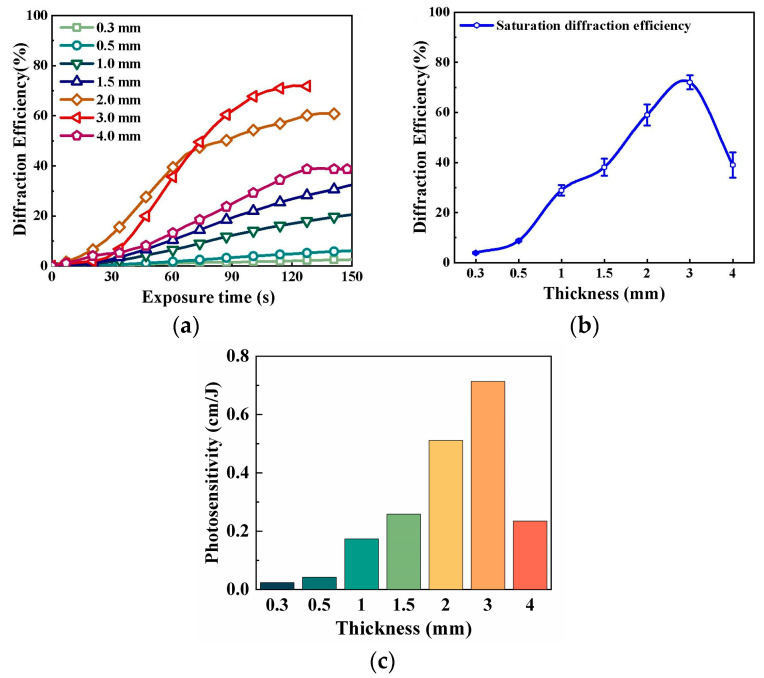
(**a**) Diffraction efficiency curves of PQ/PMMA materials with different thicknesses as a function of exposure time. (**b**) Saturation diffraction efficiency of PQ/PMMA materials with different thicknesses. (**c**) Photosensitivity of PQ/PMMA materials with different thicknesses. Reproduced with permission from ref. [78] Copyright 2024 Society of Photo-Optical Instrumentation Engineers (SPIE).

Material thickness directly impacts storage capacity and Bragg selectivity. Wang L. et al. prepared PQ/PMMA materials with thicknesses ranging from 0.3 mm to 4.0 mm [78]. The study found that the holographic performance of the material does not change monotonically with thickness. As thickness increased, the saturated diffraction efficiency showed a trend of first increasing and then decreasing (Figure 23a,c). Within the range of 1.5 mm to 3.0 mm, photosensitivity increased with thickness but decreased at 4.0 mm (Figure 23b). Comprehensive analysis indicated that under the given experimental conditions, the 3.0 mm-thick material exhibited the best saturated diffraction efficiency and photosensitivity, providing parameter guidance for achieving high-density multiplexed storage.

## 7. Conclusions and Perspectives

At last, in response to the performance limitations of PQ/PMMA photopolymer, researchers have developed a variety of effective enhancement strategies, which are summarized as follows in the Table 1:

In summary, to enhance the performance of PQ/PMMA photopolymer for collinear holographic storage, researchers have developed a series of effective strategies. These have significantly improved the properties of PQ/PMMA materials, bringing them closer to practical requirements, and have revealed deeper insights into the intrinsic relationship between the material’s microstructure and macroscopic holographic performance. By introducing comonomers or solvents like NVP, AA, NMP, and DMF, the residual monomer content was effectively increased, and the solubility limit of PQ was overcome; by doping with nanomaterials such as GO, RGO, and C_60_, not only was dispersion improved, but their unique physicochemical properties (e.g., adsorption, π-π interactions, radical initiation) were also harnessed to modulate the polymer microstructure, confirming from both positive and negative aspects that the source of the polarization response in PQ/PMMA materials is PMMA; by introducing efficient initiators or crosslinkers like TEA, POSS, and PETA, star-shaped or network macromolecular structures were constructed, accelerating photoreactions and suppressing volume shrinkage. Furthermore, the establishment of automated preparation platforms addressed material reproducibility issues, while the utilization of dark reactions provided new ideas for performance optimization at the system level.

Nevertheless, research on PQ/PMMA photopolymers still faces challenges. Future development directions may include:

1. Multi-strategy synergy for performance enhancement: Combine multiple strategies such as solubility enhancement, nanocomposite formation, and crosslinked network construction to comprehensively address issues related to sensitivity, diffraction efficiency, shrinkage, and polarization response. For example, introducing functional nanoparticles into a low-molecular-weight matrix modulated by DMF is expected to achieve superimposed breakthroughs in performance.
2. In-depth Mechanistic Understanding: Traditional characterization methods (e.g., FTIR, GPC, DSC) are mostly offline analyses performed after material preparation, making it difficult to reveal the real-time dynamic processes of photochemical reactions, molecular diffusion, and grating formation during holographic recording. Developing in situ characterization techniques, such as in situ Raman spectroscopy and in situ UV-vis absorption spectroscopy, allows simultaneous monitoring of changes in chemical functional groups and photosensitizer concentration. The combination of these techniques can provide valuable information on reaction kinetics and compositional evolution, which is crucial for deeply understanding the reaction mechanism and optimizing formulations. Furthermore, theoretical calculations (e.g., quantum chemistry, molecular dynamics) should be integrated to gain deeper insights into the interactions between dopants and the polymer matrix, photopolymerization kinetics, and the microscopic mechanisms of grating formation—particularly the mechanisms governing polarization response—in order to more effectively enhance the polarization response of the materials.
3. From “laboratory formulation” to “industrial-scale production”: While improving performance, further optimization of long-term stability and fatigue resistance is required. Most current PQ/PMMA materials are based on irreversible photopolymerization and are essentially “write-once, read-many” media. The phenomenon of NMP-PQ/PMMA achieving six rewritable cycles, discovered by Jin J. et al. [73], has opened new avenues for developing rewritable materials. However, the number of rewrite cycles and the retention of performance still need significant improvement. The main reasons for performance degradation during multiple recording cycles are the irreversible consumption of the photosensitizer PQ and the depletion of residual monomers. Future efforts could consider encapsulating the photosensitizer (or radical-generating groups) and polymerizable monomers separately in microcapsules or phase-separated structures, consuming only a portion of the active components per recording and replenishing them via thermal diffusion, thereby extending the service life. Accelerated aging tests under high temperature and high humidity have shown that introducing comonomers such as NVP can increase the thermal decomposition temperature of the material [97], thus enhancing stability. Furthermore, adding radical scavengers and antioxidants to the material could be considered to suppress dark reactions and matrix degradation induced by residual free radicals during long-term storage or repeated readout. Moreover, although the raw materials for PQ/PMMA are inherently inexpensive, some high-performance modification strategies (e.g., using POSS, fullerenes, high-purity GO) significantly increase material costs. Future development should prioritize low-concentration, high-efficiency, industrially compatible modification approaches to promote the practical application of collinear holographic storage technology.

## Figures and Tables

**Table 1:** Key Methods classification for enhancing the primary properties of PQ/PMMA materials and core improvement mechanisms.

Strategy Classification	Specific Method	Core Mechanism	Key Performance Improvement (Compared to the Original PQ/PMMA)
Matrix regulation	Doped NVP copolymer monomer [96,97]	Increase C=C content to enhance PQ solubility	Sensitivity ↑ 2×, diffraction efficiency ↑ 20%, anti-aging ↑
	Introduction of Ma-POSS/V4D4 [54,99]	Constructing star/mesh network structures	Sensitivity ↑ 5.5×, contraction rate ↓ to 0.09%
	Introduction of NMP/DMF solvents [58,73]	Reduce molecular weight, increase residual monomer	Sensitivity ↑ 6.9–9.1×, enabling repeated recording
Triggering system	Introducing AA+TEA [55,72]	Electron donors accelerate radical formation	Sensitivity ↑ 10×, detecting negative birefringence
	Introducing PETA [98]	Highly reactive cross-linking reduces the reaction energy barrier	Diffraction efficiency ↑ to 80%, with molding time ↓
Nano-doping	Introducing GO [49]	Grafting PMMA to increase PMMA content	Polarization diffraction efficiency ↑ 10×, sensitivity ↑ 3×
	Introduction of RGO (Size Effect) [56]	Controlling PMMA grafting and molecular weight	Orthogonal diffraction efficiency ↑ 3.5×, sensitivity ↑ 4.6×
	Introduction of C_60_ [51]	π-π stacking restricts PQ orientation and hinders PMMA incorporation	Intensity holography ↑, but polarization holography ↓
Process optimization	Reduce thermal polymerization time [74]	Increase residual monomer content	Effective operating time ↑ 30%, diffraction efficiency ↑
	Automated preparation [57,75]	Eliminate human error and enhance uniformity	Uniformity ↑ (error < 10%), repeatability ↑

## Data Availability

No new data were created or analyzed in this study. Data sharing is not applicable to this article.
